# Women’s Entrepreneurial Contribution to Family Income: Innovative Technologies Promote Females’ Entrepreneurship Amid COVID-19 Crisis

**DOI:** 10.3389/fpsyg.2022.828040

**Published:** 2022-03-29

**Authors:** Taoan Ge, Jaffar Abbas, Raza Ullah, Azhar Abbas, Iqra Sadiq, Ruilian Zhang

**Affiliations:** ^1^Changzhou Academy of Governance, Changzhou, China; ^2^Antai College of Economics and Management, Shanghai Jiao Tong University, Shanghai, China; ^3^Institute of Agricultural and Resource Economics, Faculty of Social Sciences, University of Agriculture, Faisalabad, Pakistan; ^4^Centre for Social Responsibility in Mining, Sustainable Minerals Institute (SMI), University of Queensland, Brisbane, QLD, Australia

**Keywords:** entrepreneurship, income contribution, women empowerment, gender, innovation technologies, COVID-19

## Abstract

Women entrepreneurs innovate, initiate, engage, and run business enterprises to contribute the domestic development. Women entrepreneurs think and start taking risks of operating enterprises and combine various factors involved in production to deal with the uncertain business environment. Entrepreneurship and technological innovation play a crucial role in developing the economy by creating job opportunities, improving skills, and executing new ideas. It has a significant impact on the income of the household. The study focused on investigating the role of women’s entrepreneurship and innovation technologies in contributing to household income in the challenging situation of the pandemic COVID-19. The paper emphasized identifying the determinants of female entrepreneurial contribution toward household income. This study collected data from selected rural and urban areas of district Faisalabad through a self-administered questionnaire. Investigators interviewed female entrepreneurs and chose them through the snowball sampling technique from a population of purposively selected female-run businesses. Interviews were conducted with women entrepreneurs to gather relevant information for the survey investigation at their workplaces and home. The effects of various factors, including age, education, family size, income from other sources, time allocated to entrepreneurial activity, firm size, and location (rural/urban) were estimated empirically using an ordered logit model. The study findings exhibited a positive and significant role of respondents’ education, family size, time allocated to entrepreneurial activities, and firm size. The survey outcomes also indicated that the contribution of entrepreneurial income to household income in the rural areas is significantly higher than that in urban areas. This study signifies that regulations against gender discrimination in public and private institutions are helpful. Besides, encouraging an environment for entrepreneurial culture among women in the country would increase family income. The study’s findings and policy implications directly link to Sustainable Development Goal (SDGs) 5 of Gender Equality (GE) and SDG 8 related to decent work and economic growth.

## Introduction

Entrepreneurship creates employment opportunities and economic development as SMEs are economic engines to the local and global economies. Entrepreneurs initiate and organize business ventures that provide critical solutions for addressing the challenges of poverty in rural areas worldwide (Sutter et al., 2019; Meyer, 2020; Bruton et al., 2021; Castellanza, 2022). Poverty alleviation remains a challenging global problem and the literature has reported a surge in entrepreneurial activities, which help reduce poverty (Fafchamps and Quisumbing, 2005; Karnani, 2007; Mair and Marti, 2009; Chatterjee et al., 2021; Castellanza, 2022). The concept of entrepreneurship was first introduced by the Franco–Irish economist Cantillon in the 18th and early 19th centuries (Jonsson, 2017). The notion of entrepreneurship comes from the French verb “entreprendre,” which is equivalent to the English concept to undertake. An entrepreneur is a person who is ready to take the risk of a new business if there is a significant chance to gain profit (Calvin, 2003). According to Penrose (1963), entrepreneurship is an activity that includes recognizing opportunities in the system of the economy. According to the views of Hisrich and Reters (2004), entrepreneurship is the process of generating something new by giving the necessary time and struggle and getting results in terms of profit, personal satisfaction, and independence.

Entrepreneurship is a common belief that it improves economic activities, family income, and social welfare. Entrepreneurial activities are also helpful in poverty reduction and achieving the Millennium Development Goals (MDGs; Mahjabeen, 2008). There has been a significant increase in entrepreneurial activities over the last three decades (Yadav and Unni, 2016). It is considered more appropriate business than employment in public or private sectors (Delmar and Gunnsson, 1997). Female participation in entrepreneurial activities is also on the rise in recent decades, and it reached around 10 percent of the global entrepreneurship activities (Saidapur and Sangeeta, 2012). Female entrepreneurship is essential for several reasons, including women empowerment (Helms, 1997), social inclusion (Altan-Olcay, 2015), economic freedom, contribution toward household income, sense of accomplishment, reducing inequalities (Kimhi, 2010), and poverty alleviation (Gu and Nie, 2021) among others.

The vision of the entrepreneurial woman in development programs is based on the assumption that women’s businesses can simultaneously create positive effects for economic development and gender equality. Accordingly, when women earn money, they are more likely than men to spend it on household needs (Nichter and Goldmark, 2009). Thereby, it facilitates poverty reduction and better quality care for children. Fostering women’s entrepreneurship is also “smart economics” because it can contribute to economic efficiency and growth (Razavi, 2012). Likewise, women become active in business and get empowered as entrepreneurship increases their control over money. According to Hashemi et al. (1996), women entrepreneurs can exercise more power in household decisions. All in all, access to monetary resources creates a “virtuous spiral” (Mayoux, 2001). As a result, women gain the ability to challenge gendered cultural practices and renegotiate social and political inequalities.

The COVID-19 crisis adversely affected developing entrepreneurs, particularly countries with limited government support (Aqeel et al., 2022; Zhou et al., 2022). Globally, vaccination is still challenging for the population (Su et al., 2021a,b). Hence, lockdown strategy and restrictions on the movement are the significant factors affecting entrepreneurial activity (Aqeel et al., 2021; Nasar et al., 2021; Yoosefi Lebni et al., 2021; Rahmat et al., 2022). It triggered unexpected crises and changed the order of routine life and the business world. In this challenging condition, the survival of the small business is at high risk (Afshan et al., 2021), with women-owned enterprises suffering the most (Kumar and Singh, 2021). All the risks associated with the business are indeed the responsibility of entrepreneurs, and some of the female entrepreneurs could not bear the business risks for long (Ribeiro et al., 2021).

Keeping in view the importance of women’s entrepreneurship for social inclusion, poverty reduction, and overall economic growth, the international development institutions, governments, NGOs, and businesses have collaborated to initiate programs to enhance women’s entrepreneurship. These programs can also help overcome gender inequality issues as it is believed that women’s economic activities can alter household power relations and encourage women’s participation in all public spheres (Altan-Olcay, 2015). Closing gender gaps in entrepreneurship can be a game-changer. Such gaps represent a lost opportunity for poverty reduction, job creation, growth, and innovation (Olsson et al., 2017; Dong et al., 2021; Aparicio et al., 2022).

There is rich literature providing insights into the determinants of entrepreneurship and its economic returns. According to the expected utility theory, individuals choose self-employment when they expect higher returns from doing so relative to wage-employment (Rees and Shah, 1986). In contrast, according to the non-pecuniary benefits theory, people select into entrepreneurship even if the expected returns are lower, searching for non-pecuniary benefits, such as being their boss (Hamilton, 2000). However, entrepreneurs are not a homogenous group of individuals, and the type of entrepreneurship engaged in may significantly affect the returns.

The literature on conventional entrepreneurship mainly concentrates on the male entrepreneur developed in the 1930s. The late 1970s observed the development of an obvious sub-domain of female entrepreneurship (Jennings and Brush, 2013). Emerging literature recommends that females play an essential part in economic development (Sarfaraz et al., 2014). More women entrepreneurs exist in emerging countries where the conventionally birth rate is very high. The law and customs of marriages have a higher influence rate if women want to become an entrepreneur (Manolova et al., 2008; Merluzzi and Burt, 2020). Females in low-income countries require strong encouragement to make their specific work atmospheres that are well suited with their children’s education and household duties at the same time. Embedding is principally vibrant for female entrepreneurship until a woman entrepreneur’s performance is sincerely made by family structure and social connections in high- and low-income countries. Different cultural backgrounds may yield different policy outcomes (Star and Yudkin, 1996).

The development of women’s entrepreneurship can help female entrepreneurs generate additional income that can be used for the sustenance of their families and improve their households’ welfare status (Thomson, 2002). In a conservative society in many developing economies, the success of female entrepreneurship has a link to or adjudged by the financial contribution for the household (Coy et al., 2011). Despite extant literature on entrepreneurship, the role of innovative technologies in promoting female-led businesses has mainly remained undiscovered while such an investigation on these factors in the pandemic times is, indeed, the need of the hour. Therefore, this research emphasized the main focus of identifying the critical components of the financial contribution of female entrepreneurship toward the household. This present paper reports on several sections. The second section provides detailed methodological approaches used in sampling, collecting the required information and analytical techniques used, the third section offers critical findings of the study, and the fourth section presents concluding remarks.

## Materials and Methods

### Study Areas and Sampling

The present research emphasized assessing the contribution of women’s entrepreneurship in household income in district Faisalabad. This study has chosen the Faisalabad district purposively. It is the home of many rural enterprises and women’s entrepreneurship. This survey gathered desired datasets from selected rural and urban areas and used self-administered questionnaire forms. The research is in line with the previous survey-based studies (Abbas et al., 2019; Mamirkulova et al., 2020; Mubeen et al., 2020,^2021^; Aman et al., 2022; Azizi et al., 2021). Investigators interviewed 150 female entrepreneurs, including 75 urban and 75 rural participants and followed survey procedure to receive data (Liu et al., 2021; Mubeen et al., 2021; Wang et al., 2021; Li et al., 2022). The study recruited respondents through the snowball sampling technique from a population of purposively selected female-run businesses. Interviews were conducted with women entrepreneurs to gather relevant information for the survey investigation at their workplaces and home. Investigators discarded two cases, one each from rural and urban areas, due to incomplete data. The rural regions included Thekri Wala, Sadhar, Dhandra, Pendra, 3 Chak, 2 Chak, Nathochak, and Shehbazpur villages. In contrast, the urban locations had Gulburge, Guru-Nanak Pura, Muhammad Pura, Ghulam Muhammadabad, Dhobi Ghat, and Anar Kali.

### Data Collection

The collected study data report on women entrepreneurs from the chosen urban and rural areas. Investigators used a survey and collected desired data through structured questionnaires, observation, and interviews (Abbas et al., 2019; Aman et al., 2019, 2022; Yoosefi Lebni et al., 2020; Abbasi et al., 2021; Hussain et al., 2021). After pre-testing, the study used a questionnaire and modified it after consultation with experts (NeJhaddadgar et al., 2020; Khazaie et al., 2021; Mohammadi et al., 2021; Soroush et al., 2021). The investigators excluded pre-tests in the final sample size. All ethical concerns were kept in mind while interviewing a respondent. Researchers obtained the prior consent of the respondents before starting the interviews and conducted the interviews in private through face-to-face meetings at their homes and workplaces. As the population for the instant study is unknown, we used the following equation to derive sample size from the study area. As the population for the instant study is unknown, we used the following equation to derive sample size from the study area:


$$n=\frac{p\left(1-p\right)Z^{2}}{e^{2.}}$$

Where, *p* = 25% (0.25), *z* = 1.96, *e* = 7% (0.07)

A total of 147 (≅150) sampled respondents.

### Data Analysis

This study incorporated various statistical techniques, including frequencies, percentages, counts, and averages, to analyze the received data statistically (Anjum et al., 2017; Moradi et al., 2021; Fu and Abbas, 2021; Mamirkulova et al., 2022). Statistics describes it as the ordered logistic regression, proportional odds, or ordinal regression. Peter McCullagh introduced this regression model for the first time dealing with ordinal dependent variables. This model deals with the responses and predicts other questions that could be quantitative (McCullagh, 1980; Baetschmann et al., 2015). This model applied an ordered logit model to determine the effect of independent variables on the dependent variable.

The general form of the ordered logit model is given below.


(1) $$Y=\beta_{0}+\beta_{1}X_{1}+\beta_{2}X_{2}+\beta_{3}X_{3}+\beta_{4}X_{4}+\beta_{5}X_{5}+\beta_{6}X_{6}+\beta_{7}X_{7}+ei$$

Where, *Y* = contribution in household income (1 = up to 25 percent, 2 = up to 50 percent, and 3 = above 50 percent)

$X_{1}$ = Age, $X_{2}$ =Education, $X_{3}$ =Family size, $X_{4}$ = Income, $X_{5}$ = Time Allocated/Week, $X_{6}$ = Entrepreneurship size, $X_{7}$ = Location

$\beta_{0}$ =Constant term

$\beta_{i}$ =Coefficient of independent variables (*i* = 1, 2, 3–7)

*e* = the error/disturbance term

The ordered logit model is also known as the proportional odds model. In the ordered logit model, there is an observed ordinal variable, Y, and an unobserved latent variable Y^*^ with various cut points. During the present study, the investigators asked respondents to indicate the percentage of financial contribution to the total household income through their entrepreneurial activities. Their responses were categorized into 1 = up to 25% contribution, 2 = 26 to 50% contribution, and 3 = above 50% contribution. Hence, the “Y” has three categories with two cutpoints in our case. The probability of an individual falling into one of the three categories. It will be subject to the following conditions.


$$Y_{i}=1\left(\mathrm{up}\;\mathrm{to}\;25\%\mathrm{contribution}\right)\mathrm{if}\;Y_{i}^{*}\;\;\mathrm{is}\leq K_{1}$$

$$Y_{i}=2\left(26\%\;\;\mathrm{to}\;50\%\right)\;\;\mathrm{if}\;\;K_{1}\leq Y_{i}^{*}\leq K_{2}$$

$$Y_{i}=3\left(\mathrm{Above}\;50\%\;\;\mathrm{Contribution}\right)\;\;\mathrm{if}\;\;Y_{i}^{*}\mathrm{is}\leq K_{3}$$

For instance, it might describe that if the score on the unobserved latent variable of Y^*^ contributed up to 25%; then, “Y” is equal to the first category. If the Y^*^ score has contributed 26 to 50%; then, “Y” is equivalent to the second category. If the Y^*^ score contributes more than 50%, “Y” equals the third category. The probabilities of each respondent for the three categories have been calculated using the following ormulas.


$$P\left(Y=1\right)=\frac{1}{1+\exp\left(Zi-K1\right)}$$

$$P\left(Y=2\right)=\frac{1}{1+\exp\left(Zi-K2\right)}-\frac{1}{1+\exp\left(Zi-K1\right)}$$

$$P\left(Y=3\right)=1-\frac{1}{1+\exp\left(Zi-K2\right)}$$

K_1_ = Cut point 1

K_2_ = Cut point 2.

## Results and Discussion

Various enterprises reported that female respondents remained engaged in their economic activities in rural and urban areas. The following table lists these business firms and the frequency and percentage of sampled female respondents who participated in the survey from each enterprise. Table 1 provides further details below.

**Table 1:** Distribution of sampled respondents in different enterprises.

Enterprises	Rural (*n* = 74)		Urban (*n* = 74)		Overall (*n* = 148)	
	Freq.	% age	Freq.	% age	Freq.	% age
Stitching	20	27.0	16	21.6	36	24.3
Beauty Parlor	4	5.4	19	25.7	23	15.5
Private School and Tuition centers	9	12.2	12	16.2	21	14.2
Agriculture value addition	6	8.1	3	4.0	9	6.1
Canteen/food distribution	3	4.0	2	2.7	5	3.4
Embroidery	9	12.2	8	10.8	17	11.5
Boutique	2	2.7	11	14.8	13	8.8
Shop	3	4.0	1	1.4	4	2.7
Poultry and Livestock	16	21.6	1	1.4	17	11.5
Making Candies	2	2.7	1	1.4	3	2.0

*Source: Survey data 2019*.

As reported both in rural and urban areas in district Faisalabad, the dominant source of economic activity is stitching. A total of 24.3% of female respondents were engaged in stitching, followed by beauty parlors. Where 15.5% of the female, primarily urban respondents, were employed. Private schools and tuition centers comprised the third-largest category in this sample, where 14.2% of the sampled female were reported to be engaged. Embroidery and Poultry and Livestock formed the fourth largest enterprise regarding the involvement of sampled females in the study area. Poultry and livestock are observed mainly in rural areas, while embroidery is evenly distributed in rural and urban areas. A total of 8.8% of sampled respondents engaged in Boutiques and 6.1% were involved in agricultural value addition. Similarly, 3.4% were engaged in food-related enterprises, 2.7% in shops and retail enterprises. Besides, 2% of respondents were making candies.

### Contribution Into Household Income

Different enterprises yield different returns. Therefore, various contributions toward household income. The following table illustrates the distribution of sampled respondents based on their contribution to family income. Table 2 given below provides further details.

**Table 2:** Distribution of sampled respondents based on their entrepreneurial contribution toward household income.

Contribution categories	Rural areas (*n* = 74)		Urban areas (*n* = 74)		Total (*n* = 148)	
	Freq.	% age	Freq.	% age	Freq.	% age
>25%	11	14.86	36	48.65	47	31.75
25–50%	23	31.08	22	29.73	45	30
<50%	40	54.05	16	21.62	56	37.84

*Source: Authors calculations form survey data*.

A general observation from the table indicates that the contribution of female entrepreneurial activities is significant in the case of rural areas where the overall household incomes are low due to lower economic opportunities in rural areas (Afrin et al., 2010). The dominant income source in the rural areas is agriculture and allied activities, where returns are relatively lower when compared with the economic activities of urban areas. The table clearly depicts this fact and illustrates that over half of the sampled rural female respondents (54%) contributed more than 50 percent toward their total household income from their entrepreneurial activities. On the other hand, the largest segment of the sampled female respondents (48.65%) contributed up to 25% toward their total household income from their entrepreneurial activities in urban areas. Overall, the contribution of entrepreneurial activities toward household income is relatively even, as depicted from the table. There are 31.75% cases where female entrepreneurs contributed up to 25 percent of the total household income. In contrast, 30% of entrepreneurs contributed between 25 percent and 50 percent toward total household income, and 37.84% contributed more than 50% toward their total household income. These figures highlight the importance of female entrepreneurship in households’ welfare and financial viability.

Various factors affect the performance and, therefore, the income from entrepreneurial activities. Table 3 provides the summary statistics of variables identified from the literature. These factors significantly affect the entrepreneurial contribution toward household income.

**Table 3:** Summary statistics of the variables.

Variables (units)	Mean	Std. deviation	Maximum	Minimum
Age (Years)	31.12	10.19	61	18
Education (Schooling years)	4.18	4.64	16	0
Family size (Members)	4.94	1.91	12	2
Income from other source (PKR^1^/Month)	57773,65	40679,91	250,000	5,000
Time allocated (Hours/Day)	5.89	2.12	13	3
Enterprise size (No. of hired Employees)	2.76	2.06	15	1

*Source: Authors calculations from survey data*.

1 *PKR is abbreviation for Pakistani Rupees (1 USD = approx. PKR 162)*.

The findings revealed that the respondents’ age ranged from 18 to 61 years, with an average of 31.12 years in this sample. Though the maximum education (schooling years) as reported during the survey was 16 years, the data indicated 4.18 average years of schooling in the study area, indicating that most of the sampled female respondents were illiterate or had lower educational attainments. The findings revealed that most female entrepreneurs started their enterprises at an early age (Powell and Kimberly, 2013). It happed to supplement their family incomes by quitting education. The average family size as reported in the surveys was 4.94 members per family with a range of 2–12 family members. Similarly, the average monthly income of the sampled households was PKR 57773.65 per month with a minimum to a maximum range of 5,000 to 250,000. The average time allocated to entrepreneurial activities by the sampled female respondents was 5.89 h per day, ranging from 3 to 13 h a day. The average enterprise size, measured by the number of hired employees, was 2.76 employees per enterprise. The minimum to a maximum range of employees was 1 to 15 employees. The surveyors found most of the micro-enterprises during the survey with up to 10 employees (Poole, 2018; Chege and Wang, 2019).

### Factors Effecting Contribution of Entrepreneurship in Household Income

An ordered logit model is used in the study to assess the impacts of various determinants on the contribution of entrepreneurship toward household income. The contribution of entrepreneurial activities in household income has been categorized into three groups, i.e., up to 25 percent contribution toward household income, 25 to 50% contribution toward household income, and above 50 percent contribution toward total household income. Table 4 shows the parameter estimates of the empirically estimated ordered logit model and the odds ratio, as given below.

**Table 4:** Factors effecting contribution of entrepreneurship in household income (*n* = 148).

Variables (units)	Coefficient	Odds ratio
Age (years)	0.022(0.021)	1.022(0.021)
Education (schooling years)	0.132^***^(0.041)	1.141^***^(0.047)
Family Size (no. of family members)	0.378^***^(0.112)	1.460^***^(0.163)
Income from other sources (PKR/Month)	−0.005(0.005)	0.995(0.005)
Time Allocated/working hours (hours/day)	0.448^***^(0.102)	1.565^***^(0.160)
Enterprise Size (No. of hired employees)	0.754^***^(0.259)	2.125^***^(0.552)
Location	−0.748^*^(0.453)	0.473^*^(0.214)
/cut1	5.625	
/cut2	7.756	
Pseudo*R*^2^	0.279	
LR chi^2^ (7)	90.31^***^	
Log Likelihood	−116.753	

Standard errors are in parenthesis. ^*^ and ^***^ represent statistical significance at 10 and 1%, respectively.

The estimated ordered logit model points to the importance of education, family size, time allocated to entrepreneurial activities, enterprise size, and location contributing to entrepreneurship activities toward total household income. Education is an investment in the human capital that benefits an entrepreneur’s performance in business survival, firm growth, the firm’s return on investment, and is likely to enhance learning and increase the problem-solving ability of an individual within a given environment (Amaral et al., 2009; Verheul et al., 2009). There is a critical role of previously acquired knowledge in intellectual performance, integration and accumulation of new knowledge, and adaptation to new situations (Weick, 1996). Previous studies estimated the returns to schooling were 6.1 percent per schooling year in developed countries and that the returns were higher for females than males (Van der Sluis et al., 2008). The findings suggest a positive and significant relationship between schooling years and entrepreneurial contribution toward household income. It indicates that increasing one schooling year will increase the chances of higher contribution (more than 50% contribution toward household income) relative to the base contribution (up to 25% contribution) by 14%. Khan et al. (2021) also found a positive impact of females’ education on entrepreneurial success.

Family size plays a crucial role in entrepreneurship performance and contributes to household income and welfare position. More family members reflect more helping hands to shoulder burdens and responsibilities in the enterprise (Abbas et al., 2019; Vernet et al., 2019; Wang and Lin, 2019; Zhao et al., 2020; Dong et al., 2021; Saridakis et al., 2021). Colombier and Masclet (2008), Sørensen (2007), and Carr and Sequeira (2007) found that children of entrepreneurs are more likely to contribute to entrepreneurial activities (Carr and Sequeira, 2007). Our findings highlighted a positive and significant impact of family size on entrepreneurial contribution toward household income. An increase of one family member will increase the chances of higher contribution by 46 percent, relative to the base category of lower contribution. Muhammad et al. (2021) found that a family consisting of 6–7 family members positively affects the household financial situation (Coy et al., 2011; Muhammad et al., 2021).

Entrepreneurs’ time is a crucial resource for these ventures (McCarthy et al., 1990), and the allocation of entrepreneurs’ time influences venture performance (Piva, 2018). Time allocated to entrepreneurial activities significantly enhances the chances of higher entrepreneur contribution toward household income. The findings suggest that increasing 1 h per day to entrepreneurial activities will increase the chances of a higher contribution of entrepreneurship toward household income by 56 percent relative to the base category of lower contribution toward household income. Our findings are consistent with Talavera et al. (2017), who also reported that more time allocation to different entrepreneurial activities enhances the performance of entrepreneurship and hence its contribution toward household income and welfare.

The size of the enterprise also affects the performance and contribution of entrepreneurship toward household income. An enterprise with more employees has higher productivity. It enables effective delegation of activities (Churchill and Lewis, 1983; Cooper et al., 1997) and leads to more leisure time or increased productivity (Verheul et al., 2009). The findings illustrate that adding one hired employee will increase the chances of higher contribution towards household income by 112 percent relative to the baseline category. The relationship between enterprise size and enterprise’s financial contribution toward household income is statistically significant.

The firm’s strategic location, which includes nearness to input and output markets, accessibility to business premises, and road network, is arguably the most crucial factor that shapes and determines the success or failure of entrepreneurs (Minai and Lucky, 2011). The performance of their business activities (Kala and Guanghua, 2010), entrepreneurship development (Dhahri and Omri, 2021), and type of product or service the firm tend to offer (Lafuente et al., 2010). Our findings suggest that the entrepreneurial contribution toward household income is higher in the case of rural areas compared to urban areas. Female entrepreneurs that contribute more than 50% to household income decreased by 52.7% if the entrepreneur belongs to urban areas. However, the finding is statistically significant only at a 10% probability level.

Age and income from other sources, in our case, have an insignificant impact on the entrepreneurial contribution toward household income. The age of the entrepreneur reflects the endowments of human capital, i.e., experience (Gimeno et al., 1997; Cowling and Taylor, 2001). Older people have more opportunities to build up relevant human capital for entrepreneurship; however, the impact of additional experience is likely to diminish with an increase in age (Verheul et al., 2009). Our findings suggest an insignificant positive effect of age on the contribution of entrepreneurship toward total household income. The value of the odds ratio indicates that keeping all other variables constant at their mean values, increasing the age of the sampled female respondents by 1 year will increase the probability of higher contribution toward household income only by 2 percent relative to the base category of lower contribution. This study findings are consistent with the literature evidence and confirmed that age positively affects entrepreneurship and income (Wang and Wong, 2004; Haley and Marsh, 2021; Olayide et al., 2021).

There is also an insignificant negative impact of income from other sources on the entrepreneurial contributions toward household income. Though financial wealth provides necessary financial resources to fuel entrepreneurial growth (Dunn and Holtz-Eakin, 2000), higher incomes from other sources tend to diminish the share of entrepreneurial contribution in household income. The finding is in line with the previous publications, Ajayi-Obe and Parker (2005), for example, argued that the availability of other sources of income is likely to reduce the preference for working hours and thereby affect productivity and performance of the enterprise.

## Conclusion

This study designed a framework based on women’s entrepreneurship and explored how entrepreneurial activities boost family income in rural and urban areas. The study findings provide valuable insights and practical implications to encourage women to initiate and run businesses. Women entrepreneurship in village cadres plays an indispensable role in solving economic inequalities and job inadequacy problems. Government and private enterprises should encourage women and provide support to stimulate the women entrepreneurial activities and business ventures of rural and urban cadres. Women in rural areas typically encounter noticeably different considerations of financial activities, which limits to financial perspective (Coleman, 2007). It usually fails to explain, predict, or stimulate the logic and mechanism of women’s entrepreneurship activities to meet labor demand. Policymakers should note the factors that constrain women from running businesses. It will boost women start-ups’ performance and make a suitable environment for entrepreneurship contribute to community poverty reduction.

Entrepreneurship has emerged as a powerful strategy to address issues related to unemployment and poverty both in developed and developing economies around the globe. Female entrepreneurship has the advantages of gender equality, women empowerment, social inclusion, economic freedom, and it contributes to household income and welfare. The study’s findings reported the significant role of women’s entrepreneurship toward household income and identified potential factors affecting the entrepreneurial contribution in household income. Entrepreneurship contribution is significantly affected by literacy, family size, time allocated to business activities, and enterprise size (Powell and Kimberly, 2013). Due to lower overall revenue in the rural areas, the entrepreneurial contribution is higher (in percentage terms) compared to urban areas. The study’s findings and its policy implications directly link to Sustainable Development Goal (SDG) 5 of Gender Equality and SDG 8 related to decent work and economic growth. The findings suggest stimulation of time investment of women in the business activities through creating awareness of the importance of relevant experience and knowledge for new venture success.

There is also a need to organize communication campaigns, particularly in rural areas, on the importance of access to formal education for girls up to higher levels and in sectors with job creation potential (non-traditional sectors). Financial literacy programs alongside management skills training for women can be proved beneficial for developing women’s entrepreneurship. The contribution of this work in the theory of organizational behavior and financial inclusion stems from the explicit roles of innovative techniques in improving entrepreneurial ability under conditions of uncertainty. The results imply emphasizing the creative options to enable entrepreneurs, particularly women, to take additional measures for the sustainability of their business. In addition, the study conclusion suggests more excellent prospects of financial inclusion for female-run enterprises that can boost their performance to generate added returns and increased employment generation, particularly among developing nations. However, additional steps would be required to provide training options for such entrepreneurs in developing countries who are deemed to be devoid of proper skills and mandatory knowledge for undertaking risky ventures that are highly vulnerable to pandemics like COVID-19.

## Data Availability Statement

The raw data supporting the conclusions of this article will be made available by the authors, without undue reservation.

## Author Contributions

All authors listed have made a substantial, direct, and intellectual contribution to the work, and approved it for publication.

## Conflict of Interest

The authors declare that the research was conducted in the absence of any commercial or financial relationships that could be construed as a potential conflict of interest.

## Publisher’s Note

All claims expressed in this article are solely those of the authors and do not necessarily represent those of their affiliated organizations, or those of the publisher, the editors and the reviewers. Any product that may be evaluated in this article, or claim that may be made by its manufacturer, is not guaranteed or endorsed by the publisher.
